# Open access for the non-English-speaking world: overcoming the language barrier

**DOI:** 10.1186/1742-7622-5-1

**Published:** 2008-01-04

**Authors:** Isaac CH Fung

**Affiliations:** 1 Department of Infectious Disease Epidemiology, Imperial College London, London, UK

## Abstract

This editorial highlights the problem of language barrier in scientific communication in spite of the recent success of Open Access Movement. Four options for English-language journals to overcome the language barrier are suggested: 1) abstracts in alternative languages provided by authors, 2) Wiki open translation, 3) international board of translator-editors, and 4) alternative language version of the journal. The *Emerging Themes in Epidemiology *announces that with immediate effect, it will accept translations of abstracts or full texts by authors as Additional files.

Editorial note:

In an effort towards overcoming the language barrier in scientific publication, ETE will accept translations of abstracts or the full text of published articles. Each translation should be submitted separately as an Additional File in PDF format. ETE will only peer review English-language versions. Therefore, translations will not be scrutinized in the review-process and the responsibility for accurate translation rests with the authors.

## The language barrier

English has become the dominant language in the global scientific community [1-3]. With English as our medium of international communication, as long as we master it, we have access to 90% of the recently published articles indexed in MEDLINE [4]. An article written in English is far more likely to be cited and has a higher impact [5-9].

Since the beginning of the MEDLINE database in 1966, the annual number and percentage of non-English journal articles indexed in it has continually decreased, from 81227 (47%) published in 1966 to 49083 (10%) in 2000 [4]. This trend applies to all non-English languages [10]. Scientists from non-English-speaking countries are under increasing pressure to publish their research outputs in English-language journals [4,8] and non-English-language journals are switching to English in order to compete internationally [3,9,11]. This is a question of efficiency: how does the scientific community maximize our returns given limited resources at our disposal?

However, as science is increasingly published in English, non-English speakers suffer a genuine disadvantage. Suggestions have been made to help non-native-English-speaking authors publish in English-language journals, including hiring native-English-speaking specialized scientific writers [12] or pairing up with 'mentors' from affluent countries with much experience in scientific publishing [13]. But this is not enough. Not only should we help our colleagues in low income countries to publish in international journals so that they can play a more active role in the global scientific community and in international policy-making, we need to empower the lay people in these countries by facilitating scientific communication to them.

Despite the laudable effort of the Open Access Movement to make publicly funded scientific research freely available to all [14], research funded by non-Anglophone governments and public funds, of which the high-quality outputs are very likely to be published in English, become less accessible to their people due to language barriers. Research participants in developing countries, who supply the raw information on which many publications are based, often will not be able to read English. Even today, many doctors in rural China are not fluent in English. How can one "make the results of a clinical trial of a new drug accessible and understandable both to doctors who might prescribe it and to people who might start taking it"[14], if they cannot read English in the first place? The dilemma of publishing in English for the global scientific research community *versus *the vernacular for the local readers, researchers and lay people alike, is a genuine problem even in an Open Access context. Although it is argued that language barrier is not the *biggest *problem in scientific communication but information overload [15], it is nevertheless a *big *problem [16]. How should we address the issue of language inequity in scientific publishing and thus the dissemination of scientific knowledge across the world [17]?

It has been suggested that translation can be encouraged by limiting the restrictive copyright statements in journals to the original language [16]. In the case of Open Access journals like the *Emerging Themes in Epidemiology*, articles are distributed under the terms of the Creative Commons Attribution License [18] and the copyright is retained by the author. As long as the original work is properly cited, the door to translation of journal articles into alternative languages is open wide.

## Abstracts in other languages?

Perhaps the first step is to translate abstracts into multiple languages. Latin American journal articles indexed in Scientific Electronic Library Online (SciELO) [19] have English abstracts in addition to their Spanish or Portuguese originals. Most Chinese language journals today require authors of original research articles to submit an English abstract. Non-English-language journals have English abstracts available to make their articles more accessible to the international scientific community, and hopefully to boost their citation rates and impact factors. But why are there so few English language journals doing it the other way round and making their articles more accessible to the non-English-speaking world?

Perhaps there are two major reasons. Editors and publishers may think that there is no demand for this, as most readers of learned journals are intellectuals who, very likely, can read English. Why then should we make a great effort for such a tiny return?

My answer is, if we believe that the lay public have a right to access the outputs of publicly funded scientific research which aims to eventually improve their quality of life [20], this applies not only to the English-speaking world, but everyone on earth. From Shanghai to São Paulo, from Hanoi to Harare, medical research of public health importance is being conducted. Scientists, medical professionals, non-governmental organization workers, and lay participants alike, should have open access to biomedical information without the language barrier. There is an ethical imperative to disseminate scientific knowledge in multiple languages, and thus mitigate the ills of the North-South inequity [16,17].

Furthermore, from the point of view of an Open Access journal, the availability of abstracts in alternative languages may increase the hit rate and download rate of its articles. By providing abstracts in alternative languages, potential readers will be able to locate relevant information through search engines using keywords in their own languages. They will then be linked to websites of Open Access journals. After reading the abstracts in their own language, interested readers can decide whether it is worth their efforts to read the whole paper in English, or seek help from teachers or translators.

However, one may wonder, even if we are persuaded to make our research outputs more accessible to the non-English-speaking peoples, how can we solve the second problem, the pragmatic one, namely the lack of resources, funding, manpower and editorial language expertise to do so? Editors may be monolingual. To hire professional translators is costly. To have abstracts available in languages of non-Roman scripts may be technically complicated. And in what languages should the abstracts be made available?

## Four options ahead

There are at least four options ahead for English-language journals to consider:

• To make abstracts available in alternative languages as an option for the authors. Since 2006, PLoS journals have encouraged authors to submit translation of their abstracts or entire articles as Supporting Information [21]. If a journal editorial board does not have the language expertise to check the accuracy of the translation, they can hold the authors responsible by declaring that this is the responsibility of the authors, not of the journals.

• To employ the idea of Wiki open editing [22,23] which allows anyone registered with the journal website to offer a translation of an abstract or even an entire article while other users can edit it. However, this is vulnerable to abuse by internet pranksters as was Wikipedia on April Fools' Day [24] and thus requires editorial monitoring.

• To recruit an international board of translator-editors, academics who are fluent in both English and their own languages and are willing to sacrifice their time and efforts to make biomedical research outputs more accessible to their fellow countrymen by translating abstracts from English into alternative languages without pay (just the same way as they volunteer to be peer reviewers of academic journals). An example is the *British Journal of Ophthalmology *[25], which offers Chinese and Portuguese translations of the abstracts of all articles in clinical and laboratory science, freely available online, with the help of only three academic editors in alternative languages: one in Hong Kong and two in São Paulo [26].

• To launch alternative language versions of a journal. An example is the *British Medical Journal *(BMJ) which provides international editions in different regions, in either English (like the West African edition) or alternative languages (Chinese, Portuguese, Romanian and Turkish). Selections of materials from the weekly BMJ relevant to local needs are presented with local news, views, commentaries and editorials [27].

## Towards multi-lingual publishing

Although we are now living in a global village where distance "shrinks" as the internet network grows, we are far from a utopia where everyone can have access to scientific literature without barriers, including the language barrier. Many scientists from the non-English-speaking world still struggle to write and publish papers in English [4,17]. Many lay people cannot read English properly [16]. The responsibility rests with us scientists and journal editors working in the English-speaking world, given the vast resources we have at hand compared to our counterparts in the developing world, to facilitate the dissemination of scientific knowledge between North and South and between the Anglophone peoples and the non-Anglophone peoples [17]. My vision of the future is that we can move beyond the first wave of the Open Access Movement towards its second wave: Multi-lingual Open Access publishing. Journals like *Revista de Saude Publica *have already adopted a bilingual online Open Access model [28]. As a Chinese proverb says, "A journey of a thousand miles begins with your first step". Therefore, the editorial board of *Emerging Themes in Epidemiology *announces that with immediate effect, our journal welcomes authors who are fluent in languages other than English to provide translations of the abstracts or full text of their articles as Additional files, submitted in PDF format. As an example, the abstract of this editorial has been translated into different languages (see Appendix). Let this be our first step to overcome the language barrier.

## Appendix: Abstracts in non-English languages

The abstract of this editorial has been translated into the following languages by the following translators (names in brackets):

• Arabic (Ms. Marilyn Chbeir and Dr. Awab Ghulam Rahim) [see Additional file 1]

• Bengali (Dr. Mohammed Aminullah Chowdhury and Mrs. Zakia Chowdhury) [see Additional file 2]

• Chinese – simplified characters (The author) [see Additional file 3]

• Chinese – traditional characters (The author) [see Additional file 4]

• Dutch (Dr. Don Klinkenberg) [see Additional file 5]

• Farsi/Persian (Dr. Awab Ghulam Rahim) [see Additional file 6]

• Filipino (Dr. Teddy Cheng) [see Additional file 7]

• French (Mr. Philip Harding-Esch) [see Additional file 8]

• German (Dr. Gesine Meyer-Rath) [see Additional file 9]

• Greek (Ms. Artemis Koukounari) [see Additional file 10]

• Hindi (Dr. Naveen Nishchal and Dr. Brajendra K. Singh) [see Additional file 11]

• Irish (Ms. Ide Cremin and Dr. Daithí Ó Murchú) [see Additional file 12]

• Italian (Ms. Delia Boccia) [see Additional file 13]

• Japanese (Dr. Naomi Seki) [see Additional file 14]

• Korean (Dr. Jae-In Ryu) [see Additional file 15]

• Latin (Mr. Toby Hudson) [see Additional file 16]

• Malay (Mr. CHONG Chun Ming) [see Additional file 17]

• Pashto (Dr. Awab Ghulam Rahim) [see Additional file 18]

• Polish (Dr. Kasia Stepniewska) [see Additional file 19]

• Portuguese – Brazilian (Dr. Cynthia Braga) [see Additional file 20]

• Romanian (Dr. Delizia Irina Chis-Ster) [see Additional file 21]

• Russian (Dr. Pavlo Minaev) [see Additional file 22]

• Shona (Dr. Zivai Mupambirei) [see Additional file 23]

• Spanish (Dr. Clarence Tam) [see Additional file 24]

• Swahili (Dr. Susan Ogada) [see Additional file 25]

• Swedish (Dr. Sabine Gabrysch and Dr. Anna Spångeus) [see Additional file 26]

• Tamil (Dr. Anto P. Rajkumar) [see Additional file 27] (requires New Kannan font for correct display of Tamil script)

• Thai (Dr. Wirichada Pongtavornpinyo) [see Additional file 28]

• Urdu (Dr. Adnan A. Khan) [see Additional file 29] (requires Noorin86 font for correct display of Urdu script)

• Vietnamese (Prof. LIN Ming-Hua and Dr. TRAN Cong Thanh) [see Additional file 30]

## Competing interests

The author(s) declare that they have no competing interests.

## Authors' contributions

ICHF conceived the idea of this editorial, wrote it and read and approved its final manuscript.

## Funding

The author receives no funding for this editorial.

The author is one of the managing editors of *Emerging Themes in Epidemiology*.

## Supplementary Material

Additional File 1 Abstract in Arabic. Click here for file

Additional File 2 Abstract in Bengali. Click here for file

Additional File 3 Abstract in Chinese (Simplified characters). Click here for file

Additional File 4 Abstract in Chinese (Traditional characters). Click here for file

Additional File 5 Abstract in Dutch. Click here for file

Additional File 6 Abstract in Farsi (Persian). Click here for file

Additional File 7 Abstract in Filipino. Click here for file

Additional File 8 Abstract in French. Click here for file

Additional File 9 Abstract in German. Click here for file

Additional File 10 Abstract in modern Greek. Click here for file

Additional File 11 Abstract in Hindi. Click here for file

Additional File 12 Abstract in Irish. Click here for file

Additional File 13 Abstract in Italian. Click here for file

Additional File 14 Abstract in Japanese. Click here for file

Additional File 15 Abstract in Korean. Click here for file

Additional File 16 Abstract in Latin. Click here for file

Additional File 17 Abstract in Malay. Click here for file

Additional File 18 Abstract in Pashto. Click here for file

Additional File 19 Abstract in Polish. Click here for file

Additional File 20 Abstract in Portuguese (Brazilian). Click here for file

Additional File 21 Abstract in Romanian. Click here for file

Additional File 22 Abstract in Russian. Click here for file

Additional File 23 Abstract in Shona. Click here for file

Additional File 24 Abstract in Spanish. Click here for file

Additional File 25 Abstract in Swahili. Click here for file

Additional File 26 Abstract in Swedish. Click here for file

Additional File 27 Abstract in Tamil. Click here for file

Additional File 28 Abstract in Thai. Click here for file

Additional File 29 Abstract in Urdu. Click here for file

Additional File 30 Abstract in Vietnamese. Click here for file

## References

[B1] Garfield E (1977). English – An International language for science, The Information Scientist, Dec 76. Essays of an Information Scientist.

[B2] Ofori-Adjei D, Antes G, Tharyan P, Slade E, Tamber PS (2006). Have Online International Medical Journals Made Local Journals Obsolete?. PLoS Medicine.

[B3] Garfield E (1989). The English language- The lingua Franca of international science. Scientist.

[B4] Loria A, Arroyo P (2005). Language and country preponderance trends in MEDLINE and its causes. J Med Libr Assoc.

[B5] Garfield E (1977). Le-Nouveau-Defi-Americain.1. Current Contents.

[B6] Garfield E (1976). [La science francaise est-elle trop provinciale?] (SCIENCE IN FRANCE – TOO PROVINCIAL). Recherche.

[B7] Garfield E, Welljamsdorof A (1990). Language use in international research - A citation analysis. Annals of the American Academy of Political and Social Science.

[B8] Garfield E (1988). French research- Citation analysis indicates trends are more than just a slip of the tongue. Current Contents.

[B9] Garfield E, Welljamsdorof A (1992). The microbiology literature- languages of publication and their relative citation impact. Fems Microbiology Letters.

[B10] Waheed AA (2001). Scientists turn to journals in English. Scientific World Journal.

[B11] Puliselic L, Petrak J (2006). Is it enough to change the language? A case study of Croatian biomedical journals. Learned Publishing.

[B12] Victora CG, Moreira CB (2006). [North-South relations in scientific publications: editorial racism?]. Rev Saude Publica.

[B13] Freeman P, Robbins A (2006). The publishing gap between rich and poor: the focus of AuthorAID. J Public Health Policy.

[B14] Eisen MB, Brown PO, Varmus HE (2004). PLoS Medicine – A Medical Journal for the Internet Age. PLoS Medicine.

[B15] Garfield E (1983). The Foreign Language Barrier: Problems in Scientific Communication. Nature.

[B16] Green CW (2000). North and South: bridging the information gap. Lancet.

[B17] Horton R (2000). North and South: bridging the information gap. Lancet.

[B18] Creative Commons Attribution License. http://creativecommons.org/licenses/by/2.0.

[B19] Scientific Electronic Library Online. http://www.SciELO.org/.

[B20] The Alliance for Taxpayer Access. http://www.taxpayeraccess.org.

[B21] The PLoS Medicine Editors (2006). Ich Weiss Nicht Was Soll Es Bedeuten: Language Matters in Medicine. PLoS Medicine.

[B22] What is Wiki. http://www.wiki.org/wiki.cgi?WhatIsWiki.

[B23] Wiki (Wikipedia entry). http://en.wikipedia.org/wiki/Wiki.

[B24] Wikipedia braces itself for April Fools' Day. http://www.guardian.co.uk/g2/story/0,,2044243,00.html.

[B25] British Journal of Ophthalmology. http://bjo.bmj.com/.

[B26] Kwok AHK, Pereira DS, Bhisitkul RB (2004). Found in translation. Br J Ophthalmol.

[B27] International Editions of the British Medical Journal. http://group.bmj.com/group/rights-licensing/translations/internationaleditions.

[B28] Goldbaum M (2003). [Revista de Saude Publica (Journal of Public Health) in electronic English version]. Rev Saude Publica.

