# Supplementary material for: Open access for the non-English-speaking world: overcoming the language barrier
Source: Emerg Themes Epidemiol. 2008 Jan 4;5:1. doi: 10.1186/1742-7622-5-1 (PMC2268932; doi:10.1186/1742-7622-5-1)
Supplement: Additional File 1 — Abstract in Arabic. [file 1742-7622-5-1-S1.pdf]

Arabic / العربية

افتتاحية

الوصول الحرّ للعالم غير الناطق بالانكليزية: تجاوز عائق اللغة

الكاتب: Isaac Chun-Hai FUNG

خلاصة

تسلّط هذه الافتتاحية الضوء على مشكلة عائق اللغة في التواصل العلمي بالرغم من نجاح "حركة الوصول الحرّ" الأخير. وتقترح أربعة خيارات على المجلات الصادرة باللغة الانكليزية لتجاوز هذا العائق:

1) خلاصات يؤمّنها الكتّاب بلغات بديلة،

2) الترجمة الحرّة بواسطة Wiki،

3) مجلس دولي للمترجمين والمحررين،

4) إصدار نسخة من المجلة بلغة بديلة.

تعلن مجلة Emerging Themes in Epidemiology انها تستقبل ترجمات لخلاصات أو نصوص يرسلها الكتّاب على شكل ملفات إضافية.
