# Supplementary material for: Open access for the non-English-speaking world: overcoming the language barrier
Source: Emerg Themes Epidemiol. 2008 Jan 4;5:1. doi: 10.1186/1742-7622-5-1 (PMC2268932; doi:10.1186/1742-7622-5-1)
Supplement: Additional File 2 — Abstract in Bengali. [file 1742-7622-5-1-S2.pdf]

Bengali/বাংলা

সম্পাদকীয় প্রবন্ধ

ইংরেজী ভাষা ভাষী নয় এমন লোকদের জন্য অবাদ প্রবেশ পথ : ভাষাজনিত বাধার অতিক্রম

গ্রন্থকার: ইসাক চুন-হাই ফাং Isaac Chun-Hai FUNG

সারাংশ :

সাম্প্রতিক বৈজ্ঞানিক যোগাযোগের ক্ষেত্রে অবাদ প্রবেশের আন্দোলনের সাফল্যতার স্বত্ত্বেও এই সম্পাদকীয় প্রবন্ধ ভাষাজনিত বাধার সমস্যাকে সকলের দৃষ্টিগোচর করে। ইংরেজী ভাষার সাময়িক পত্রিকায় এই বাধা অতিক্রমের চারটি উপায় উল্লেখ করা হয় :

১) লেখক বিভিন্ন ভাষায় প্রবন্ধের সারংশ সরবারহ করবে।

২) উইকি (Wiki) খোলা অনুবাদ।

৩) আন্তর্জাতিক অনুবাদ - সম্পাদকীয় সমিতি

৪) সাময়িক পত্রপত্রিকাগুলোতে বিভিন্ন ভাষায় প্রকাশনা।

``রোগবিস্তার বিদ্যার উদ্ভুত বিষয়বস্তু `` প্রকাশনা ঘোষনা করেছে যে অবিলম্বে লেখকের রচনার সম্পূর্ণ বা সারাংশের অনুবাদ তারা গ্রহন করবে৷
