# Supplementary material for: Open access for the non-English-speaking world: overcoming the language barrier
Source: Emerg Themes Epidemiol. 2008 Jan 4;5:1. doi: 10.1186/1742-7622-5-1 (PMC2268932; doi:10.1186/1742-7622-5-1)
Supplement: Additional File 3 — Abstract in Chinese (Simplified characters). [file 1742-7622-5-1-S3.pdf]

Simplified Chinese / 简体中文

编者语

为非英语世界提供公开取阅：克服语言障碍

作者：冯俊熙　(Isaac Chun-Hai FUNG)

综述

这篇编者语指出了在近年来公开取阅运动的成功下，科学交流仍然存在着语言障碍。四种克服语言障碍的可行方案被提出来供各英文期刊参考：１）由作者提供综述的其他语言版本，２）以维基方式公开让人翻译，３）国际性的翻译编辑委员会，和４）期刊的其他语言版本。《流行病学中的新近主题》(*Emerging Themes in Epidemiology*) 宣布：由即日起，它将会接受作者以附加档案的形式，提供综述或全文翻译。
