# Supplementary material for: Open access for the non-English-speaking world: overcoming the language barrier
Source: Emerg Themes Epidemiol. 2008 Jan 4;5:1. doi: 10.1186/1742-7622-5-1 (PMC2268932; doi:10.1186/1742-7622-5-1)
Supplement: Additional File 4 — Abstract in Chinese (Traditional characters). [file 1742-7622-5-1-S4.pdf]

Traditional Chinese / 繁體中文

編者語

爲非英語世界提供公開取閱：克服語言障礙

作者：馮儁熙　(Isaac Chun-Hai FUNG)

綜述

這篇編者語指出了在近年來公開取閱運動的成功下，科學交流仍然存在著語言障礙。四種克服語言障礙的可行方案被提出來供各英文期刊參考：１）由作者提供綜述的其他語言版本，２）以維基方式公開讓人翻譯，３）國際性的翻譯編輯委員會，和４）期刊的其他語言版本。《流行病學中的新近主題》(*Emerging Themes in Epidemiology*) 宣佈：由即日起，它將會接受作者以附加檔案的形式，提供綜述或全文翻譯。
