# Supplementary material for: Open access for the non-English-speaking world: overcoming the language barrier
Source: Emerg Themes Epidemiol. 2008 Jan 4;5:1. doi: 10.1186/1742-7622-5-1 (PMC2268932; doi:10.1186/1742-7622-5-1)
Supplement: Additional File 5 — Abstract in Dutch. [file 1742-7622-5-1-S5.pdf]

Dutch / Nederlands

Editorial

Open Access voor de niet-Engelstalige wereld: het slechten van de taalbarrière

Auteur: Isaac Chun-Hai FUNG

Samenvatting

Dit editorial stelt het probleem van taalbarrières in wetenschappelijke communicatie centraal, dat ondanks het huidige succes van de Open Access Movement nog altijd bestaat. Er worden vier opties voor Engelstalige tijdschriften voorgesteld om de taalbarrière te slechten: 1) samenvattingen in alternatieve talen, verschaft door de auteurs, 2) vertalingen via Wiki open translation, 3) internationale commissies van vertalers en redacteuren en 4) alternatieve versies van tijdschriften in andere talen. Emerging Themes in Epidemiology kondigt aan dat het met ingang van heden vertalingen van samenvattingen of volledige teksten door de auteurs zal accepteren in de vorm van electronische bijlagen.
