# Supplementary material for: Open access for the non-English-speaking world: overcoming the language barrier
Source: Emerg Themes Epidemiol. 2008 Jan 4;5:1. doi: 10.1186/1742-7622-5-1 (PMC2268932; doi:10.1186/1742-7622-5-1)
Supplement: Additional File 6 — Abstract in Farsi (Persian). [file 1742-7622-5-1-S6.pdf]

فارسی / Farsi (Persian)

سرمقاله/Editorial

Open Access for the non-English-speaking world: Overcoming the language barrier.

دسترسی آزاد برای گوینده های که به زبان انگلیسی تسلد ندارد: از بین بردن مانع زبانی.

نویسنده/Author: Isaac Chun-Hai FUNG

خلاصه/Abstract

این سرمقاله مشکلات موانع زبانی در مفاهمه علمی رابا وجود حرکت آزاد موفقیت های اخیر برملا میسازد. چهار بدیل جهت ازبین بردن موانع زبانی در ژورنالهای لسان انگلیسی پشنهاد گردیده.

1- خلاصه های تهیه شده در لسانهای متبادل توسط مولفین(نویسنده ها).

2- ترجمه باز Wiki

3- بورد بین المللی مولفین و مترجمین

4- ترجمه ژورنال به زبان متبادل

پدیدار شدن موضوعات نو در اپیدیمولوژی (Emerging themes in Epidemiology) جهت اجرات سریع اعلام میدارد که !

ترجمه خلاصه و یا مکمل متن توسط نویسنده گان بقسم یک فایل اضافی قابل قبول خواهد بود.
