# Supplementary material for: Open access for the non-English-speaking world: overcoming the language barrier
Source: Emerg Themes Epidemiol. 2008 Jan 4;5:1. doi: 10.1186/1742-7622-5-1 (PMC2268932; doi:10.1186/1742-7622-5-1)
Supplement: Additional File 7 — Abstract in Filipino. [file 1742-7622-5-1-S7.pdf]

Filipino / Pilipino / Tagalog

Editoryal

Pagpalawak ng Partisipasyon sa mga Hindi Maka-komprende ng Ingles: Pag-tanggal ng Limitasyon sa Lengguahe

May-akda: Isaac Chun-Hai FUNG

Abstrakto

Pinapaliwanag nitong editoryal ang mga problema ng limitasyon ng salita sa lengguahe, kahit sa kasalukuyang tagumpay ng 'Pagpalawak sa Partisipasyon' (Open Participation Movement / Open Access Movement).

Apat na opsyon para sa diurnal na Ingles ang salita para sa Pag-tanggal ng Limitasyon sa Lengguahe. Heto ang nasusonod:

1) Abstrakto ng panibagong lengguahe ng may-akda,

2) Pabukas na translasyon galing sa 'Wiki',

3) Translasyon galing sa kasapi ng Pinunong Internasyonal translasyon-editoryal,

at 4) Panibong lengguahe ng diurnal.

Ang diurnal 'Bagong Tema ng Epedimiyolohiya' (Emerging Themes in Epidemiology) ay importanteng pinapa-alam sa lahat na ito ay tumatanggap ng translasyon ng abstrakto o ng buong artikulo ng mga may-akda para sa adisyonal na literatura.
