# Supplementary material for: Open access for the non-English-speaking world: overcoming the language barrier
Source: Emerg Themes Epidemiol. 2008 Jan 4;5:1. doi: 10.1186/1742-7622-5-1 (PMC2268932; doi:10.1186/1742-7622-5-1)
Supplement: Additional File 8 — Abstract in French. [file 1742-7622-5-1-S8.pdf]

French / Français

Editorial

Accès Ouvert pour le monde non-anglophone: surmonter la barrière linguistique

Auteur: Isaac Chun-Hai FUNG

Résumé

Cet éditorial souligne le problème de la barrière linguistique dans la communication scientifique en dépit du récent succès du mouvement de l'Accès Ouvert. Quatre options sont proposées afin que les revues scientifiques anglophones puissent surmonter la barrière linguistique: 1) des résumés dans d'autres langues fournies par les auteurs. 2) la traduction libre par wiki. 3) un conseil international de traducteurs-rédacteurs. 4) publier une version de la revue scientifique dans une autre langue. Emerging Themes in Epidemiology annonce que, dès maintenant, les traductions des résumés ou des textes complets des auteurs seront acceptées sous forme de fichiers supplémentaires.
