# Supplementary material for: Open access for the non-English-speaking world: overcoming the language barrier
Source: Emerg Themes Epidemiol. 2008 Jan 4;5:1. doi: 10.1186/1742-7622-5-1 (PMC2268932; doi:10.1186/1742-7622-5-1)
Supplement: Additional File 9 — Abstract in German. [file 1742-7622-5-1-S9.pdf]

German / Deutsch

Leitartikel

Open Access für die nicht-englischsprachige Welt: Sprachbarrieren überwinden.

Autor: Isaac Chun-Hai FUNG

Zusammenfassung

Dieser Leitartikel beleuchtet das Problem der Sprachbarrieren im wissenschaftlichen Austausch, das trotz des derzeitigen Erfolgs der Open-Access-Bewegung bestehen bleibt. Vier Möglichkeiten zum Überwinden der Sprachbarrieren in englischsprachigen wissenschaftlichen Zeitschriften werden vorgeschlagen: 1) vom Verfasser verantwortete Zusammenfassungen in anderen Sprachen, 2) offene Übersetzung nach dem Wiki-Prinzip , 3) Übersetzung durch ein internationales Kommittee von Herausgebern, und 4) Herausgabe anderssprachiger Versionen der wissenschaftlichen Publikation. Emerging Themes in Epidemiology kündigt an, dass ab sofort vom Verfasser angefertigte Übersetzungen von Zusammenfassungen oder des gesamten Artikels als Dateianhang akzeptiert werden.
