# Supplementary material for: Open access for the non-English-speaking world: overcoming the language barrier
Source: Emerg Themes Epidemiol. 2008 Jan 4;5:1. doi: 10.1186/1742-7622-5-1 (PMC2268932; doi:10.1186/1742-7622-5-1)
Supplement: Additional File 10 — Abstract in modern Greek. [file 1742-7622-5-1-S10.pdf]

Modern Greek / Νέα Ελληνικά

Άρθρο

Ανοιχτή Πρόσβαση για τον κόσμο που δεν μιλάει Αγγλικά: ξεπερνώντας τα εμπόδια ξένης γλώσσας.

Συντάκτης: Isaac Chun-Hai Fung

Περίληψη

Αυτό το άρθρο επισημαίνει το πρόβλημα των εμποδίων λόγω γλώσσας που υπάρχουν στην επιστημονική επικοινωνία παρά την πρόσφατη επιτυχία του Κινήματος της Ανοιχτής Πρόσβασης (Open Access Movement). 4 επιλογές ώστε να ξεπεραστούν τα εμπόδια λόγω γλώσσας, για εφημερίδες Αγγλικής γλωσσας, προτείνονται: 1) περιλήψεις σε εναλλακτικές γλώσσες παρέχονται απο συγγραφείς, 2) ουίκι (Wiki) ανοιχτή επικοινωνία, 3) διεθνής επιτροπή μεταφραστών-συντακτών, και 4) μετάφραση του επιστημονικού περιοδικού σε άλλη γλώσσα. Το επιστημονικό περιοδικό «Υπάρχοντα θέματα στην Επιδημιολογία» (Emerging Themes in Epidemiology) αναγγέλλει οτι με άμεσο σκοπό, θα δέχεται μεταφράσεις περιλήψεων ή πλήρη κείμενα από συντάκτες σαν επιπλέον αρχεία.
