# Supplementary material for: Open access for the non-English-speaking world: overcoming the language barrier
Source: Emerg Themes Epidemiol. 2008 Jan 4;5:1. doi: 10.1186/1742-7622-5-1 (PMC2268932; doi:10.1186/1742-7622-5-1)
Supplement: Additional File 11 — Abstract in Hindi. [file 1742-7622-5-1-S11.pdf]

Hindi / हिन्दी

सम्पादकीय

गैर-आँग्ल भाषियों के लिए मुक्त स्रोत : भाषायी बंधन से मुक्ति

लेखक : आईज़क चुन-हाय फुँग (Isaac Chun-Hai FUNG)

सारांश

यह सम्पादकीय विज्ञान के संचार में भाषायी बंधन की समस्या को बखूबी उजागर करता है जो ग़त वर्षों में आयी मुक्त स्रोत क्रांति की सफलता के बावज़ूद वर्तमान है। आँग्ल पत्रिकाओं के भाषायी बंधन को समाप्त करने के लिए चार विकल्प सुझाए गये हैं:

1. लेखकों द्वारा सारांश को अन्य भाषाओं में उपलब्ध कराना,
2. विकी द्वारा अनुवाद उपलब्ध होना,
3. अनुवादक सम्पादकों के अंतर्राष्ट्रीय समूह का होना, एवं
4. पत्रिकाओं का अन्य भाषाओं में उपलब्ध होना।

इमर्जिंग थीम्स इन एपिडेमियोलॉजी (Emerging Themes in Epidemiology), यह घोषणा करता है कि तत्काल प्रभाव से लेखकों द्वारा उपलब्ध कराये गये सारांश अथवा पूरे पाठ्य का अनुवाद को अतिरिक्त फ़ाइल के रूप में स्वीकार किया जायेगा।
