# Supplementary material for: Open access for the non-English-speaking world: overcoming the language barrier
Source: Emerg Themes Epidemiol. 2008 Jan 4;5:1. doi: 10.1186/1742-7622-5-1 (PMC2268932; doi:10.1186/1742-7622-5-1)
Supplement: Additional File 12 — Abstract in Irish. [file 1742-7622-5-1-S12.pdf]

Irish / Gaeilge

Eagarfhocal *(editorial)*

**Open Access dóibh siúd nach labhraíonn Béarla : Ag sárú constacaí teangeolaíochta**

Úadar: Isaac Chun-Hai FUNG

Achomaireacht *(abstract)*

In anneoin dul chun cinn an "Open Access Movement", tugann an t-eagarfhocal seo 'súil eile' dúinn ar fhadbhanna agus ar chonstacaí teangeolaíochta a bhaineann le cumarsáid eolaíochta. Tá ceithre rogha molta ag irisí Béarla chun bacanna teanga a shárú:

1) achomairí i dteangacha eile léirithe ag na húdair,

2) aistriúchán oscailte "Wiki"

3) bord idirnáisiúnta d'eagarthóirí-aistreoirí

4) leagan an iris i dteanga eile

Fógraíodh "Emerging themes in Epidemiology", go nglacfaí láithreach le haistriúcháin na n-achomairí ó théacasanna iomlána údar, mar chomhaid breise.
