# Supplementary material for: Open access for the non-English-speaking world: overcoming the language barrier
Source: Emerg Themes Epidemiol. 2008 Jan 4;5:1. doi: 10.1186/1742-7622-5-1 (PMC2268932; doi:10.1186/1742-7622-5-1)
Supplement: Additional File 13 — Abstract in Italian. [file 1742-7622-5-1-S13.pdf]

Italian / Italiano

Editorial

Libero accesso alle publicazioni per il mondo non anglofono: come abbattere le barriere linguistiche

Autore: Isaac Chun-Hai FUNG

Abstract

Sull’onda del recente successo del Movimento “Open Acess” (*ndt*. “Accesso libero”), questo editoriale affronta il problema delle barriere linguistiche nella comunicazione scientifica. Alle riviste in lingua inglese, vengono suggerite 4 possibilita’ per il superamento di suddette barriere: 1) fornire la possibilita’ agli autori di inviare abstracts in lingue diverse dall’inglese. 2) consentire la possibilita’ di Wiki open translation, 3) formare dei comitati di traduttori internazionali all’interno della redazione delle riviste scientifiche, e 4) creare versioni di riviste scientifiche scritte in lingua diversa dall’inglese. In linea con cio’, Emerging Themes in Epidemiology (*ndt*. Temi Emergenti in Epidemiologia) annuncia che – con effetto immediato - accettera’, in aggiunta alla versione in inglese, documenti contenenti la versione in lingua originale di abstracts o di articoli interi.
