# Supplementary material for: Open access for the non-English-speaking world: overcoming the language barrier
Source: Emerg Themes Epidemiol. 2008 Jan 4;5:1. doi: 10.1186/1742-7622-5-1 (PMC2268932; doi:10.1186/1742-7622-5-1)
Supplement: Additional File 14 — Abstract in Japanese. [file 1742-7622-5-1-S14.pdf]

Japanese / 日本語

論説

非英語圏の門戸を開く：学術分野における言語バリアーをいかに克服するか

文責　馮儁熙 (Isaac Chun-Hai FUNG)

要旨

本論説は、近年の門戸解放運動（Open Access Movement）の成功にかかわらず、未だ学術分野に存在する言語バリアーの問題を取り上げたものである。本論説は英文雑誌の抱える言語バリアー解消に、１）著者による非英語言語で書かれた要旨の提供、　２）ウィキペディア（Wiki）の翻訳機能の活用、　３）国際翻訳―編集委員会の設定、　４）非英語言語バージョンの雑誌の出版、という４つの方法を提案している。今回本誌（Emerging Themes in Epidemiology）は、言語バリアー解消に迅速な効果があるとして、非英語言語で書かれた要旨もしくは全文の全訳の補足ファイル添付を承諾すると、発表した。
