# Supplementary material for: Open access for the non-English-speaking world: overcoming the language barrier
Source: Emerg Themes Epidemiol. 2008 Jan 4;5:1. doi: 10.1186/1742-7622-5-1 (PMC2268932; doi:10.1186/1742-7622-5-1)
Supplement: Additional File 15 — Abstract in Korean. [file 1742-7622-5-1-S15.pdf]

Korean / 한국어 (韓國語)

논설

비영어권에 대한 자유로운 접근을 위해: 언어의 장벽을 극복

저자: 馮儁熙 (Isaac Chun-Hai FUNG)

초록

이번 논설은 최근의 자유로운 접근에 대한 성공적인 움직임에도 불구하고 언어장벽으로 인한 과학적 소통의 어려움에 대해 이야기하고자 한다. 영어권 학술지의 언어장벽을 극복하기 위해 다음의 네 가지 방법이 추천된다. 1) 저자가 여타의 언어로 초록제출, 2) 위키(Wiki)사전의 자동번역, 3) 국제적인 번역가능 편집자 위원회 설립, 4) 학술지의 다양한 언어로의 발행. 역학의 최신주제(Emerging Themes in Epidemiology)학술지의 경우 이러한 조치를 즉각 실행할 것이며 저자가 제출한 다른 언어로 번역된 초록이나 전문을 추가파일형태로 승인하기로 하였다.
