# Supplementary material for: Open access for the non-English-speaking world: overcoming the language barrier
Source: Emerg Themes Epidemiol. 2008 Jan 4;5:1. doi: 10.1186/1742-7622-5-1 (PMC2268932; doi:10.1186/1742-7622-5-1)
Supplement: Additional File 16 — Abstract in Latin. [file 1742-7622-5-1-S16.pdf]

Classical Latin / Lingua Latina

Littera Commentariensis

Via aperta pro loquendis linguis perigrinis: quo modo quasi murus obscuritatis deleri potest.

Auctor: Isaac Chun-Hai FUNG

Abstractum

haec littera commentariensis confirmat linguae non intelligatae difficilitatem quae perstat contra quod nuper perfecit collegium nomine via aperta, id est ei qui student ad comprehensionem facilem omnibus (Open Access Movement). eis libellis, qui lingua in anglica scripti sunt, ad delendum huius quasi muri hae quattuor optiones proponantur: (I) abstracta aliis in linguis ab auctoribus praeberi; (II) translationem per beneficium nomine uiki gratuite suppeditari; (III) collegium quoddam statui eorum qui texta inter linguas transferunt atque ad praedicanda praeparant; (IV) editionem alia in lingua faci. nuntiant editores libellum studiorum rerum novarum de morbis (Emerging Themes in Epidemiology) extemplo uel editiones abstractorum ab auctoribus translatas uel texta integra uolumina adiecta recepturum esse.
