# Supplementary material for: Open access for the non-English-speaking world: overcoming the language barrier
Source: Emerg Themes Epidemiol. 2008 Jan 4;5:1. doi: 10.1186/1742-7622-5-1 (PMC2268932; doi:10.1186/1742-7622-5-1)
Supplement: Additional File 17 — Abstract in Malay. [file 1742-7622-5-1-S17.pdf]

Malay / Bahasa Melayu

Editorial

Akses terbuka untuk dunia bukan berbahasa Inggeris: Mengatasi batasan bahasa.

Pengarang: Isaac Chun-Hai FUNG

Abstrak

Editorial ini menekankan tentang masalah batasan bahasa dalam komunikasi saintifik di sebalik kejayaan Gerakan Akses Terbuka akhir-akhir ini. Empat pilihan untuk jurnal-jurnal bahasa Inggeris bagi mengatasi batasan bahasa disarankan:

1) abstrak dalam bahasa-bahasa alternatif disediakan oleh para penulis,
2) terjemahan terbuka Wiki,
3) lembaga penterjermah-editor antarabangsa, dan
4) jurnal versi bahasa alternative.

Emerging Themes in Epidemiology mengumumkan akan menerima terjemahan abstrak-abstrak atau teks-teks penuh oleh para penulis sebagai fail tambahan dengan serta merta.
