# Supplementary material for: Open access for the non-English-speaking world: overcoming the language barrier
Source: Emerg Themes Epidemiol. 2008 Jan 4;5:1. doi: 10.1186/1742-7622-5-1 (PMC2268932; doi:10.1186/1742-7622-5-1)
Supplement: Additional File 18 — Abstract in Pashto. [file 1742-7622-5-1-S18.pdf]

Pashto/ پشتو

Editorial/سریزه

Open Access for the non-English-speaking world: Overcoming the language barrier.

د نړی د هغو ویونکو لپاره اسانه لاسرسی چه انګلیسی نه کاروی

لیکوال/Author: Isaac Chun-Hai FUNG

لنديز/Abstract

دا سر مقاله د ازادی لاس رسی د یون سره سره په علمی پوهاوی کی د ژبنیو خندونو ستونزی را برسیره وی.

په انګلیسی ژبه کی د ژورنالونو د ژبنیو حندونو د له منحه ورلو لپاره حلور وراندیزونه شویدی.

1- د لیکوالانو پواسطه په نورو ژبو کی د لندیزونو برابرول

2- Wikiآزاده ژباره

3- د لیکوالانو او ژبارونکو بین المللی بورد

4- په بله ژبه کی د ژورنال ژباره

په اپیدمیولوژی کی د نویو موضوعاتو را پیدا کیدل(Emerging themes in Epidemiology) د بیرنی کرنی په موخه د لیکوالانو په مت په اضافی دول د لندیزونو یا بشپر متن زباره د منلو ور بولی.
