# Supplementary material for: Open access for the non-English-speaking world: overcoming the language barrier
Source: Emerg Themes Epidemiol. 2008 Jan 4;5:1. doi: 10.1186/1742-7622-5-1 (PMC2268932; doi:10.1186/1742-7622-5-1)
Supplement: Additional File 19 — Abstract in Polish. [file 1742-7622-5-1-S19.pdf]

Polish / język polski

Wstępniak

Otwarty dostęp dla nie-angielsko-językcznego świata: pokonanie bariery językowej

Autor: Isaac Chun-Hai FUNG

Skrót

Artykuł podkreśla problem bariery językowej w komunikacji naukowej pomimo niedawnego sukcesu Open Access Movement. Proponuje angielsko-językcznym pismom cztery sposoby na pokonanie tej bariery: 1) skróty w innych językach przesyłane przez autorów, 2) Wiki open translation, 3) miedzynarodowa rada tłumaczy-edytorów, 4) alternatywne wersje językowe pism. Emerging Themes in Epidemiology ogłasza że z efektem natychmiastowym będzie przyjmować tłumaczenia skrótów lub całych tekstów od autorów jako Additional files.
