# Supplementary material for: Open access for the non-English-speaking world: overcoming the language barrier
Source: Emerg Themes Epidemiol. 2008 Jan 4;5:1. doi: 10.1186/1742-7622-5-1 (PMC2268932; doi:10.1186/1742-7622-5-1)
Supplement: Additional File 20 — Abstract in Portuguese (Brazilian). [file 1742-7622-5-1-S20.pdf]

Brazilian Portuguese / Português brasileiro

Editorial

Acesso gratuito de trabalhos em língua não inglesa: ultrapassando as barreiras de comunicação

Autor: Isaac Chun-Hai FUNG

Resumo

Este editorial destaca as barreiras da comunicação em linguagem científica mesmo com o sucesso recente do “Open Acess Movement”. Com vistas à superação de tais dificuldades são sugeridas quatro estratégias dirigidas às revistas científicas de língua inglesa: 1) Aceitação de resumos em outras línguas, além da versão original em inglês; 2) Trabalhos traduzidos utilizando o “software” aberto “Wiki open”, 3) Organização de comitês internacionais de editores-tradutores e, eventualmente, a 4) criação de uma versão em língua não inglesa da revista. Dando suporte a esta idéia, a revista Temas Emergentes em Epidemiologia comunica que, a partir de agora, estará aceitando versões adicionais de resumos ou artigos completos em língua não inglesa submetidos pelos autores.
