# Supplementary material for: Open access for the non-English-speaking world: overcoming the language barrier
Source: Emerg Themes Epidemiol. 2008 Jan 4;5:1. doi: 10.1186/1742-7622-5-1 (PMC2268932; doi:10.1186/1742-7622-5-1)
Supplement: Additional File 21 — Abstract in Romanian. [file 1742-7622-5-1-S21.pdf]

Romanian / Limba Română

Editorial

**Bariera lingvistică, acces deschis, publicații ştiințifice, traducere.**

Autor: Isaac Chun-Hai FUNG

Rezumat

Amploarea comunicării ştiințifice internaționale interdisciplinare cunoaşte o evoluție fără precedent. Pe fondul globalizării, limba engleză este dominantă în toate domeniile de activitate. In vederea depăşirii barierei lingvistice, sugerăm următoarele patru opțiuni: 1) rezumate în limbi alternative furnizate de către autori, 2) folosirea mijloacelor de traducere web de tip Wiki, 3) crearea unui comitet internațional de traducători ştiințifici, 4) publicarea unei ediții alternative a revistelor sau jurnalelor ştiințifice într-o alta limbă. Revista *Emerging Themes in Epidemiology* informează cititorii că în curând traducerile rezumatelor sau a publicațiilor vor fi acceptate ca fişiere suplimentare.
