# Supplementary material for: Open access for the non-English-speaking world: overcoming the language barrier
Source: Emerg Themes Epidemiol. 2008 Jan 4;5:1. doi: 10.1186/1742-7622-5-1 (PMC2268932; doi:10.1186/1742-7622-5-1)
Supplement: Additional File 22 — Abstract in Russian. [file 1742-7622-5-1-S22.pdf]

Russian / Русский

Редакционная статья

Открытый доступ для неанглоязычного мира: преодолевая языковой барьер

Автор: Isaac Chun-Hai FUNG

Реферат

В данной редакционной статье идет речь о проблеме языкового барьера в научном общении, которая имеет место несмотря на недавний успех движения За Открытый Доступ. С целью преодоления языкового барьера для англоязычных журналов предложены четыре подхода: 1) использование рефератов на нескольких языках (перевод обеспечивается авторами) ; 2) открытый перевод Wiki; 3) международный совет редакторов-переводчиков; 4) публикация журналов на других языках. Emerging Themes in Epidemiology обьявляет, что с настоящего времени будет принимать рефераты статей или полные переводы статей в виде дополнительных файлов.
