# Supplementary material for: Open access for the non-English-speaking world: overcoming the language barrier
Source: Emerg Themes Epidemiol. 2008 Jan 4;5:1. doi: 10.1186/1742-7622-5-1 (PMC2268932; doi:10.1186/1742-7622-5-1)
Supplement: Additional File 23 — Abstract in Shona. [file 1742-7622-5-1-S23.pdf]

Shona / Shona

Zvinyorwa

Kupa vanhu vemunyika dzisingatuare chirungu mukana wekuvana zvinyorwa zviri mumitauro iyo vanonzwisiswa: Kuedza kukunda kusadyidzana nekunzwisisana nekuda kwekusiyana kwemitauro.

Munyori: Isaac Chun-Hai FUNG

Chinyorwa

Chinangwa chehinyorwa icho kuedza kuburitsa matambudziko ekusanzwana anokonzerwa nemitauro inoshandiswa muzvinyorwa zvesaenzi (science) zvisinei kuti Access Movement iri kurudzira kuvanikwa kwezvinyorwa izvi nemunhu vose pachena. Kuti dambudziko remitauro iri rikundwe, pane kurudziro ina dziri kupiwa kumapato anonyora nekubudisa zvinyorwa zvakanyorwa nechirungu (English language journals) kuti akunde matambudziko ayo anovanikwa nevanhu vanoda kuverenga zvinyorwa izvi asi vasingakwanise nekuda kwemitauro vakasiyana.

Chekutanga (1) vanyori ngavakuridzirwe kupa zvinyorwa zviri mumitauro yakasiyana-siyana.

Chechipiri (2) Ngakuve nekuturikirwa pachena kwemitauro yakasiyana-siyana.

Chechitatu (3) Ngakuumbwe bazi revaturikiri vemitauro nevaongorori vezvinyorwa vemitauro iyoyi.

Chechina (4) Ngakuvewo bato rema ”Journal” rinomirira kubudisa zvinyorwa zvakanyorwa nemitauro yakasiyana-siyana iyoyi chete chete.

Bato rinonzi “Emerging Themes in Epidemiology” rakabudisa kuti, pamusoro pezvinyorwa zvechirungu izvo rinobudisa, rava kutambirawo nhanganyaya (abstracts) nezvinyorwa (full texts) zvakanyorwa nemutauro upi zvavo kubva kuvanyori.
