# Supplementary material for: Open access for the non-English-speaking world: overcoming the language barrier
Source: Emerg Themes Epidemiol. 2008 Jan 4;5:1. doi: 10.1186/1742-7622-5-1 (PMC2268932; doi:10.1186/1742-7622-5-1)
Supplement: Additional File 24 — Abstract in Spanish. [file 1742-7622-5-1-S24.pdf]

Spanish / Español

Editorial

"Open Access" para el mundo no anglófonico: Superando la barrera linguística

Autor: Isaac Chun-Hai FUNG

Resumen

En este editorial se destaca el problema de la barrera linguística a la comunicación científica a pesar del reciente éxito del movimiento "Open Access". Se sugieren cuatro opciones para revistas publicadas en la lengua inglesa: 1) resúmenes proporcionados por los autores en idiomas alternativos, 2) traducción pública por medio de "wiki", 3) formación de un cuadro internacional de editores-traductores, y 4) version segundaria de la revista en lengua alternativa. Emerging Themes in Epidemiology anúncia con efecto inmediato que acepta traducciones de resúmenes o artículos completos sometidos por autores como material suplementario.
