# Supplementary material for: Open access for the non-English-speaking world: overcoming the language barrier
Source: Emerg Themes Epidemiol. 2008 Jan 4;5:1. doi: 10.1186/1742-7622-5-1 (PMC2268932; doi:10.1186/1742-7622-5-1)
Supplement: Additional File 25 — Abstract in Swahili. [file 1742-7622-5-1-S25.pdf]

Swahili / Kiswahili

Tahariri

Njia wazi kwa nchi zisizosungumza kiingereza:
Kuvuka kikwazo katika lugha

Mtunzi: Isaac Chun-Hai FUNG

Nadharia

Tahariri hii utasisitiza shida ya kikwezo katika lugha haswa katika kisayansi ya mawasiliano ijapokuwa kuna mafanikio ya hapo wali ya kwenda mbele. Maoni manne yamependekezwa katika kiingereza – jarida ya lugha kuvuka kikwazo katika lugha:

1) nadharia ya lugha yenye hairi au badala kutolewa na waandishi,
2) tafsiri ya wazi ya WIKI,
3) kamati ya kimataifa ya wakalimani na waandishi,
4) toleo la jarida ya lugha badala.

Mada inajitokeza katika epidemiology na inataangaza wazi kuanzia sasa hivi kuwa itapokea tafsiri ya nadharia au dhana au maandishi kamili ya waandishi kama jalada ya ziada.
