# Supplementary material for: Open access for the non-English-speaking world: overcoming the language barrier
Source: Emerg Themes Epidemiol. 2008 Jan 4;5:1. doi: 10.1186/1742-7622-5-1 (PMC2268932; doi:10.1186/1742-7622-5-1)
Supplement: Additional File 26 — Abstract in Swedish. [file 1742-7622-5-1-S26.pdf]

Swedish / Svenska

Ledare

Open Access för den icke-engelsk språkiga världen: Hur tar man sig över språkbarriären?

Författare: Isaac Chun-Hai FUNG

Abstract

Den här ledaren uppmärksammar problemet med språkbarriären inom den vetenskapliga kommunikationen trots den senaste tidens framgångar genom Open Access rörelsen. Fyra möjligheter föreslås för engelska tidskrifter för att övervinna språkbarriären: 1) författarna skriver sammanfattningar på alternativa språk, 2) Öppen översättning enligt ”Wiki principen”, 3) ett internationellt råd bestående av redaktörer/översättare, och 4) icke-engelsk version av tidskriften.
Emerging Themes in Epidemiology tillkännager härmed att den från och med nu accepterar bilagor med författarens översättningar av sammanfattningar eller kompletta texter.
