# Supplementary material for: Open access for the non-English-speaking world: overcoming the language barrier
Source: Emerg Themes Epidemiol. 2008 Jan 4;5:1. doi: 10.1186/1742-7622-5-1 (PMC2268932; doi:10.1186/1742-7622-5-1)
Supplement: Additional File 27 — Abstract in Tamil. [file 1742-7622-5-1-S27.pdf]

TAMIL / jkpo;

jiyaq;fk;

Mq;fpyk; Ngrhj ehLfSf;fhd midtUk; mZFk; epiy: nkhopj;jilfis Kwpabj;jy;

Mrphpah;: ISAAC CHUN-HAI FUNG

fUj;Jr; RUf;fk;:

midtUk; mZFk; epiy ,af;fj;jpd; rk;gj;jpa ntw;wpf;F khwhf mwptpay; jftY; ghpkhw;wj;jpy; nkhopj;jilfs; ngUk; rthyhf tpsq;Ftij ,ej; jiyaq;fk; Rl;bf;fhl;Lfpw;J. Mq;fpy nkhop gj;jpupiffs; nkhopj;jilfis Kwpabg;gjw;fhd ehd;F topKiwfs; ,q;F Kd;nkhopag;gl;Ls;sd,

1) Mrphpah;fs; jq;fsJ fl;Liufspd; fUj;Jr;RUf;fq;fis gpw nkhopfspy; toq;f Ntz;Lk;.

2) tpf;fp jpwe;j epiy nkhop ngah;g;G

3) nkhop ngah;g;ghsh;fs; kw;Wk; jpUj;jpa ikg;ghsh;fSf;fhd rh;tNjr mikg;G.

4) gpwnkhop tbtq;fspy; gj;jphpiffis ntspapLjy;.

Emerging themes in Epidemiology Mrphpah;fspd; fUj;JrRUf;fq;fs; kw;Wk; KOg;gbtq;fspd; nkhopngah;g;Gfis $Ljy; Nfhg;Gfshf VwWf;nfhs;Snkd cldb nray; jpl;lj;Jld; mwptpf;fpd;wJ.
