# Supplementary material for: Open access for the non-English-speaking world: overcoming the language barrier
Source: Emerg Themes Epidemiol. 2008 Jan 4;5:1. doi: 10.1186/1742-7622-5-1 (PMC2268932; doi:10.1186/1742-7622-5-1)
Supplement: Additional File 28 — Abstract in Thai. [file 1742-7622-5-1-S28.pdf]

Thai / ภาษาไทย

บทบรรณาธิการ

บุคคลที่ไม่ได้ใช้ภาษาอังกฤษในการสื่อสาร ก็สามารถใช้ Open Access ในการรับรู้ข้อมูลต่างๆได้

ผู้เขียน: Isaac Chun-Hai FUNG

บทคัดย่อ

บรรณาธิการเน้นให้เห็นถึงปัญหาของภาษาในการสื่อสารทางวิทยาศาสตร์ ถึงแม้ว่า Open Access จากสื่อต่างๆจะประสบความสำเร็จอย่างมาก 4 แนวทางแนะนำสำหรับวารสารภาษาอังกฤษในการแก้ไขปัญหาของภาษาในการสื่อสาร คือ: 1) การเตรียมคำนำ ในภาษาอื่นๆ โดยผู้เขียน 2) การใช้ระบบ Wiki ในการแปลจากภาษาหนึ่งไปยังอีกภาษาหนึ่ง 3) การจัดตั้งคณะกรรมการกลุ่มล่ามและบรรณาธิการนานาชาติ 4) การจัดภาษาเลือกให้แก่วารสารนั้นๆ กลุ่มวารสาร Emerging Themes in Epidemiology ได้ประกาศว่านับแต่นี้ไป ทางกลุ่มจะจัดตั้งข้อมูลที่เป็นคำนำ และเนื้อหาที่ได้รับการแปลจากผู้เขียนแล้ว ให้เป็นข้อมูลเสริมของระบบ
