# Supplementary material for: Open access for the non-English-speaking world: overcoming the language barrier
Source: Emerg Themes Epidemiol. 2008 Jan 4;5:1. doi: 10.1186/1742-7622-5-1 (PMC2268932; doi:10.1186/1742-7622-5-1)
Supplement: Additional File 29 — Abstract in Urdu. [file 1742-7622-5-1-S29.pdf]

Urdu /اردو

Editorial اداریہ

Open Access for the non-English-speaking world: Overcoming the language barrier.

غیر انگریزی بولتی د* کے لئے کھلی پہنچ: لسانی پ # یوں کو عبور کرنے کے راستے

Author: Isaac Chun-Hai FUNG

مصنف: آئزک - چن ہای فنگ

Abstract ا

یہ اداریہ سائنسی تبادلا ء خیال میں حالیہ کھلی پہنچ کی مہم (Open Access Movement) کے * وجود ھونے والی ز* نی اور ترجمہ سے متعلق دشواریوں تو اجاگر کرتا ہے۔ انگریزی میں شائع ہونے والے سائنسی .+ وں کے لئے لسانی دشواریوں کو حل کرنے کے چار راستے ہیں۔ ۱) مصنف خودا (abstract) دوسری ز* ن میں مہیا کریں۔ ۲) Wiki open ترجمہ۔ ۳) بین الاقوامی مترجم جما (۔ ۴) دوسری ز* ن میں اشا $ ۔ Emerging Themes in Epidemiology اعلان کرتا ہے کہ وہ مصنف کا مہیا کردہ دV ز* ن میں ا بطور اظافی file منظور کرے گا۔
