# Supplementary material for: Open access for the non-English-speaking world: overcoming the language barrier
Source: Emerg Themes Epidemiol. 2008 Jan 4;5:1. doi: 10.1186/1742-7622-5-1 (PMC2268932; doi:10.1186/1742-7622-5-1)
Supplement: Additional File 30 — Abstract in Vietnamese. [file 1742-7622-5-1-S30.pdf]

Vietnamese / tiếng Việt

Lời tòa soạn

MỞ RỘNG TIẾP CẬN CHO CỘNG ĐỒNG KHÔNG NÓI TIẾNG ANH: VƯỢT QUA RÀO CẢN NGÔN NGỮ

Tác giả: Isaac Chun-Hai FUNG

Tóm tắt:

Bài viết này nêu bật những vấn đề về rào cản ngôn ngữ trong thông tin khoa học cho dù có sự thành công gần đây của Phong trào tiếp cận mở rộng.
Có bốn tùy chọn được gợi ý giúp cho những tạp chí viết bằng tiếng Anh vượt qua rào cản ngôn ngữ:
1). Tác giả viết bài tóm tắt bằng ngôn ngữ khác được chọn
2). Bản dịch Wiki mở
3). Ủy ban quốc tế về biên phiên dịch, và
4). Phiên bản bằng ngôn ngữ được chọn khác của tạp chí
Tạp chí *Những chủ đề nổi bật trong Dịch tễ học* (Emerging Themes in Epidemiology) thông báo rằng ngay từ nay, tạp chí sẽ chấp nhận những phiên bản dịch bài tóm tắt hoặc bài toàn văn của tác giả được gửi đính kèm.
